# Effects of phlebotomy-induced reduction of body iron stores on metabolic syndrome: results from a randomized clinical trial

**DOI:** 10.1186/1741-7015-10-54

**Published:** 2012-05-30

**Authors:** Khosrow S Houschyar, Rainer Lüdtke, Gustav J Dobos, Ulrich Kalus, Martina Broecker-Preuss, Thomas Rampp, Benno Brinkhaus, Andreas Michalsen

**Affiliations:** 1 Department of Internal Medicine, Kliniken Essen-Mitte, University Duisburg-Essen, Essen, Germany; 2 Department of Biometry, Karl und Veronica Carstens-Foundation, Essen, Germany; 3 Institute of Transfusion Medicine, Charité- University Medical Centre, Berlin, Germany; 4 Department of Endocrinology and Division of Laboratory Research, University Hospital Essen, Essen, Germany; 5 Institute of Social Medicine, Epidemiology and Health Economics, Charité-University Medical Centre, Berlin, Germany; 6 Immanuel Hospital Berlin, Department of Internal and Integrative Medicine, Berlin, Germany, Königstrasse 63, 14109 Berlin, Germany

## Abstract

**Background:**

Metabolic syndrome (METS) is an increasingly prevalent but poorly understood clinical condition characterized by insulin resistance, glucose intolerance, dyslipidemia, hypertension, and obesity. Increased oxidative stress catalyzed by accumulation of iron in excess of physiologic requirements has been implicated in the pathogenesis of METS, but the relationships between cause and effect remain uncertain. We tested the hypothesis that phlebotomy-induced reduction of body iron stores would alter the clinical presentation of METS, using a randomized trial.

**Methods:**

In a randomized, controlled, single-blind clinical trial, 64 patients with METS were randomly assigned to iron reduction by phlebotomy (n = 33) or to a control group (n = 31), which was offered phlebotomy at the end of the study (waiting-list design). The iron-reduction patients had 300 ml of blood removed at entry and between 250 and 500 ml removed after 4 weeks, depending on ferritin levels at study entry. Primary outcomes were change in systolic blood pressure (SBP) and insulin sensitivity as measured by Homeostatic Model Assessment (HOMA) index after 6 weeks. Secondary outcomes included HbA1c, plasma glucose, blood lipids, and heart rate (HR).

**Results:**

SBP decreased from 148.5 ± 12.3 mmHg to 130.5 ± 11.8 mmHg in the phlebotomy group, and from 144.7 ± 14.4 mmHg to 143.8 ± 11.9 mmHg in the control group (difference -16.6 mmHg; 95% CI -20.7 to -12.5; *P *< 0.001). No significant effect on HOMA index was seen. With regard to secondary outcomes, blood glucose, HbA1c, low-density lipoprotein/high-density lipoprotein ratio, and HR were significantly decreased by phlebotomy. Changes in BP and HOMA index correlated with ferritin reduction.

**Conclusions:**

In patients with METS, phlebotomy, with consecutive reduction of body iron stores, lowered BP and resulted in improvements in markers of cardiovascular risk and glycemic control. Blood donation may have beneficial effects for blood donors with METS.

**Trial registration:**

ClinicalTrials.gov: NCT01328210

Please see related article: http://www.biomedcentral.com/1741-7015/10/53

## Background

Metabolic syndrome (METS), a condition characterized by insulin resistance, glucose intolerance, dyslipidemia, hypertension, and obesity [1], affects approximately a quarter of the population in the USA [2] and is becoming increasingly prevalent in Europe [3]. The pathogenesis of METS is incompletely understood, but recent studies have suggested that oxidative stress catalyzed by accumulation of iron in excess of physiologic requirements may be contributory [4].

Previous findings have indicated an association between accumulated iron and the components of METS, including hypertension and diabetes mellitus. Serum ferritin levels correlate with hypertensive retinopathy [5], and clinical hypertension is characterized by a higher prevalence of increased iron stores [6]. In two Danish population studies, the hemochromatosis genotype and increased transferrin saturation were associated with increased risk of requirement for antihypertensive medication [7]. A positive association between iron stores and insulin resistance or diabetes mellitus has been found in numerous epidemiologic studies [8-12]. Furthermore, both, ferritin and transferrin were shown to be significantly associated with the presence of the METS and its components [13,14]. Notably, increased ferritin levels may be a determinant for METS in postmenopausal women but not in premenopausal women [15]. It was further suggested that iron overload may be crucial for non-alcoholic fatty liver disease (NAFLD) in METS [16], and raised ferritin levels were found to be independent predictors of vascular damage in NAFLD and METS [17].

Similarly, there is recent evidence that reduction of body iron stores may improve the symptoms of METS. Iron-chelating agents and blood donation can prevent the development of diabetes in iron overload [18,19]. Depleting iron stores in type 2 diabetes by phlebotomy may favorably increase insulin sensitivity in carriers of the HFE mutation [20] and in patients with diabetes [21]. In earlier studies, repeated phlebotomy resulted in decreases in serum glucose and blood lipids [22]. In patients with non-alcoholic steatohepatitis, blood-letting also led to decreased insulin concentrations [23]. Moreover, it was reported that a low-iron diet positively influences cardiovascular risk in type 2 diabetes [24]. Finally, in an uncontrolled observational study on 15 patients with essential hypertension resistant to a triple drug regimen, repeated phlebotomy resulted in a pronounced reduction in blood pressure (BP) [25]. Iron-mediated oxidative stress may modulate vascular tone [26,27], and hepcidin, a key iron regulatory peptide, correlates with vascular damage in METS [28]

Western populations have a high prevalence of raised iron stores [29], thus if iron reduction can beneficially affect METS, this would have considerable public-health significance, as well as being beneficial for the health of the donor in certain conditions.

As the effects of iron reductive therapy in METS have not been previously addressed systematically, we designed a randomized clinical trial to measure the effects of phlebotomy on BP, insulin sensitivity, and cardiovascular risk factors in patients with METS.

## Methods

This randomized controlled trial was performed in a single centre (Kliniken-Essen-Mitte, an academic teaching hospital of the University Duisburg-Essen). Patients were enrolled between June and December 2008, and intervention and follow-up were completed in March 2009. The study was approved by the Ethics Committee of the Medical Faculty of the University Hospital Essen and is registered at ClinicalTrials.gov, NCT01328210. Informed consent was obtained from all patients.

### Participants

Patients aged 25 to 70 years old with presumed METS were recruited through press advertisements and general (family) practices. Patients were required to have three or more of the following criteria: 1) abdominal adiposity (waist circumference ≥1020 mm (men) or ≥880 mm (women)); 2) low levels of high-density lipoprotein cholesterol (HDL-C): ≤40 mg/dL (men) or ≤50 mg/dL (women)); 3) hypertriglyceridemia (≥150 mg/dL); 4) raised BP (≥130/85 mmHg); and 5) impaired glucose homeostasis (fasting plasma glucose ≥110 mg/dL). Enrollment criteria were reviewed in a screening telephone call, and confirmed by examination at the first study visit. Exclusion criteria included: 1) clinically significant other organic disease including malignancy; 2) history of hemochromatosis or presence of the Cys282Tyr mutation; 3) history of drug or alcohol misuse 4); history of disturbances in iron balance (for example,, iron overload or deficiency); and 5) anemia (hemoglobin < 12 g/dL).

Of the 113 patients that expressed interest in participating, 72 were invited to the clinic for more detailed investigation. Based on the results, 64 patients were included in the study. The population was generally middle-aged, predominantly female, and overweight population. Pre-existing diabetes was present in 38%, and nearly all had hypertension (Table 1).

**Table 1:** Clinical baseline characteristics of the study population

	Treatment group (n = 33)	Control group (n = 31)	*P *value
Age, years	60 ± 6	57 ± 11	0.52
Gender, F/M	13/20	14/17	0.88
Weight, kg	95.0 ± 18.9	92.6 ± 16.1	0.60
Body mass index, kg/m^2^	32.8 ± 5.5	32.5 ± 5.6	0.88
Waist circumference, cm	109.0 ± 11.3	108.9 ± 13.5	0.98
Type 2 diabetes, n (%)	10 (30)	14(45)	0.22
Drug intake, n (%)	31 (94)	24 (77)	0.06
History of hypertension, n (%)	32 (97)	31 (100)	0.33
History of smoking, n (%)	2 (6)	7 (23)	0.06

Data a mean ± SD unless indicated otherwise.

### Randomization

The 64 patients were randomly allocated to either the iron-reduction group (n = 33) or the control group (n = 31). Baseline characteristics were balanced between groups. The patients were randomized by means of a non-stratified block randomization method with randomly varying block lengths based on the 'ranuni' pseudo-random number generator of the SAS/Base^® ^statistical software (SAS Inc., Cary NC, USA). For each patient, the biostatistician prepared sealed, sequentially numbered, opaque envelopes containing the treatment assignments. Each time a patient fulfilled all enrolment criteria, the study physician opened the lowest numbered envelope to reveal that patient's assignment. Allocation of treatment was not blinded. Data were collected by trained blinded study personnel.

All patients received standard medical care as determined by their individual requirements. All participants were instructed to continue their usual treatment with medication, and were specifically advised to maintain their usual diet and physical activity, and to abstain from any other new treatments for METS.

### Intervention

#### Iron-reduction group

The phlebotomy intervention consisted of removal of two volumes of blood: one at entry to the study and one at day 28. Phlebotomy was performed while patients lay in the supine position. The skin was disinfected, then blood was collected via the cubital arm vein. At the first phlebotomy session, 300 ml of blood were removed. At the second calibrated blood removal, the volume removed varied in accordance with the entry level of ferritin concentration: (250 ml for patients with ferritin < 90 ng/ml, 350 ml with 90 to 200 ng/ml, and 500 ml with > 200 ng/mL).

#### Control group

Patients allocated to the control group received no specific treatment, but were offered phlebotomy at the end of the 6-week study period to ensure study compliance (waiting list design).

#### Outcome measurements

Physical measurements were taken in a quiet room while participants were in the fasting state. They were asked to refrain from smoking or caffeine for at least 60 minutes before their appointment time. BP measurements were standardized for cuff size, position, and time of day. After 10 minutes of quiet rest, two seated measurements of BP on the non-dominant arm and the heart rate (HR) were recorded with an automatic sphygmomanometer (Dynamap, Criticon, Norderstedt, Germany). At each assessment, two readings, taken with an interval of 5 minutes, were averaged to obtain the BP.

For laboratory investigations, a blood sample was drawn at baseline and at 6 weeks. An additional blood sample was collected in the bloodletting group at 4 weeks. Insulin sensitivity was estimated by the homeostasis model assessment (HOMA) index, and calculated as fasting plasma glucose (mmol/l) × serum insulin (μU/ml) ÷ 25.

Blood count and assays for blood lipids, HbA1c, ferritin and iron were performed using standard methods. Serum concentrations of insulin and high-sensitivity C-reactive proten (hs-CRP) were measured by immunonephelometry (BNiI-nephelometer, Siemens, Fernwald, Germany), and adiponectin concentrations were measured by radioimmunoassay (DRG Instruments, Marburg, Germany) at the Central Laboratory of University Hospital Essen.

Physical activity and nutritional habits were assessed by standardized self-reports and diaries. Adverse events (AEs) were monitored by diary and at the final study visit by interview.

There were two main outcome parameters in this study: change in SBP and change in HOMA index, from week 0 (pre-treatment) to week 6.

### Sample size determination and statistical analysis

We expected an effect size of 0.7 for the HOMA index and calculated that a sample size of 64 patients would be needed to give a power of β = 80% by means of a two-sided *t*-test with α = 5%. This approximates the effect of iron reduction on insulin sensitivity as measured by an intravenous insulin tolerance test in patients with type 2 diabetes patients given in a previous trial [21], in which a standardized effect of *d *= 0.78 was verified. Given the assumption that the intravenous insulin tolerance test might be more sensitive than the HOMA index, we considered that a hypothetical estimated effect size of 0.5 would yield a sample size of n = 130. No data from controlled trials were available for the estimate of the effect of phlebotomy on BP, but we expected an effect size of greater than 0.5 for this primary outcome. Finally, sample size calculation was based on an expected effect size of *d *= 0.7 with 64 patients.

All statistical analyses were performed using the SAS statistical analysis package (version 9.2; SAS Inc., Cary, NC, USA). They were carried out on an intention-to-treat basis including all randomized patients irrespective of their adherence to the study protocol. Missing data were multiply imputed using Markov chain Monte Carlo methods [30]. For each outcome parameter, this gave a total of 20 complete data sets,. These were each separately analyzed by univariate analysis of covariance (ANCOVA), which included group and baseline values as covariates. Finally, the results were adequately combined to produce overall effect size estimates, 95% confidence intervals (CI) and *P *values. To avoid multiple statistical errors, the *P *values for both primary outcome parameters were adjusted in accordance with the Bonferroni-Holm procedure [31].

## Results

**Figure 1:**
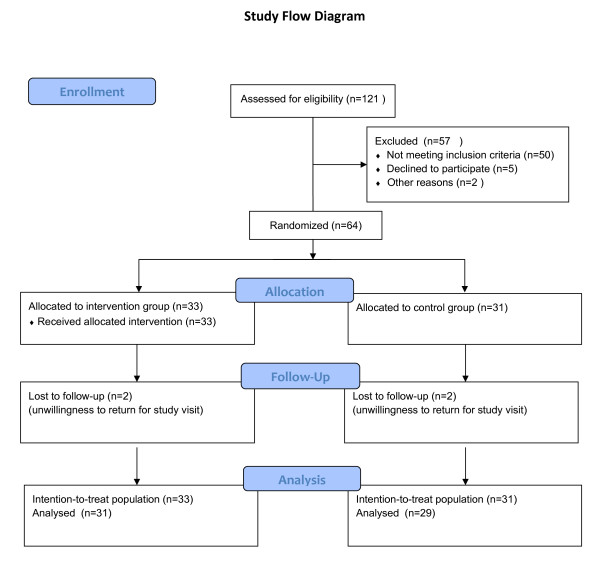
**Study flow chart**. Numbers of patients who were enrolled and included in the analysis.

Two patients in each group withdrew during the course of the study course because they were unable to return for follow-up. These patients reported during a telephone interview that they were satisfied with the study procedures and had not experienced any AEs (Figure 1). Hence, 29 patients in the control group and 31 in the treatment group completed the study.

In the iron reduction group, all patients received two phlebotomies in accordance with the study protocol. Mean hemoglobin decreased from 14.3 ± 1.2 at baseline to 13.3 ± 1.1 mg/dl after 6 weeks, and similarly, mean serum ferritin concentration decreased from 188.3 ± 212.4 to 104.6 ± 132.5 mg/dl. Medication was not modified during the study. Lifestyle habits including the amount of exercise and type and quantity of food intake, as assessed by self-reports, remained unchanged. Accordingly, mean body mass index and waist circumference remained unchanged in both groups throughout the study.

### Primary outcome measures

#### Blood pressure

The iron-reduction had a lowering of BP (Table 2) compared with the control group. After 6 weeks, the reduction in SBP was 18.3 ± 10.5 mmHg in the phlebotomy group and 0.2 ± 7.7 mmHg in the control group, resulting in a group difference of -16.5 mmHg (95% CI -20.6 to -12.3; *P *< 0.001). Hence, at study end, 25 patients (81%) of the control group were classified as hypertensive (BP ≥140/90 mmHg) compared with only 13 patients (39%) in the phlebotomy group.

**Table 2:** Primary and secondary outcomes with group differences for change after treatment

	Treatment group (n = 33)		Control group (n = 31)		Between-group difference^a^		
				
	Baseline (n = 33)	Week 6 (n = 31)	Baseline (n = 31)	Week 6 (n = 29)	**Diff**.	95% CI	*P *value
Primary outcomes							
SPB, mmHg	148.5 ± 12.3	130.5 ± 11.9	144.7 ± 14.4	143.8 ± 11.9	-16.5	-20.6 to 12.3	< 0.001
HOMA index	4.8 ± 7.2	3.6 ± 2.7	4.5 ± 3.8	4.1 ± 3.6	-0.7	-2.1 to 0.6	0.28
Secondary outcomes							
Glucose, mg/dl	110.7 ± 29.4	98.5 ± 24.0	109.1 ± 34.4	107.3 ± 33.6	-13.3	-18.8 to -8.1	< 0.001
HbA1c, %	5.56 ± 0.61	5.36 ± 0.58	5.84 ± 1.24	5.72 ± 1.24	-0.19	-0.29 to -0.08	< 0.001
Insulin, μU/ml	15.4 ± 17.7	14.2 ± 8.9	16.7 ± 12.7	15.2 ± 11.6	-1.2	-5.4 to 3.0	0.580
Adiponectin, μg/ml	9.15 ± 3.40	9.83 ± 5.00	8.75 ± 4.00	9.40 ± 4.40	0.24	-0.90 to 1.39	0.68
hs-CRP, mg/dl	0.32 ± 0.44	0.33 ± 0.39	0.30 ± 0.37	0.31 ± 0.38	0.01	-0.05 to 0.07	0.80
DBP, mmHg	93.2 ± 6.8	83.7 ± 5.7	90.8 ± 6.6	90.0 ± 8.6	-8.2	-10.7 to -5.7	< 0.001
Heart rate, beats/min	72.1 ± 8.2	70.0 ± 5.8	74.2 ± 9.5	76.2 ± 8.4	-5.7	-8.1 to -3.2)	< 0.001
Ferritin, ng/ml	188.3 ± 212.4	104.6 ± 132.5	173.2 ± 132.9	149.4 ± 124.9*	-74.2	101.6 to -46.8	< 0.001
Iron, ng/ml	100.6 ± 34.2	75.8 ± 28.5	100.8 ± 26.2	102.8 ± 36.9*	-27.1	-41.1 to -13.2	< 0.001
Triglycerides, mg/dl	154.0 ± 66.7	158.0 ± 64.7	204.6 ± 120.0	178. ± 70.7*	-2.9	-31.4 to 25.6	0.84
Cholesterol, mg/dl	208.4 ± 36.5	212.2 ± 38.6	211.0 ± 37.7	206.4 ± 36.4*	3.1	-6.7 to 12.8	0.54
HDL-C, mg/dl	55.9 ± 15.0	58.9 ± 17.9	54.2 ± 16.5	55.0 ± 16.1*	3.0	-0.3 to 6.3	0.07
LDL-C, mg/dl	131.2 ± 35.8	123.5 ± 35.9	130.7 ± 32.8	125.1 ± 29.1*	-4.9	-11.8 to 2.0	0.16
LDL/HDL ratio	2.48 ± 0.88	2.21 ± 0.80	2.56 ± 0.82	2.43 ± 0.82	-0.23	-0.38 to -0.08	< 0.01

Abbreviations: DBP, diastolic blood pressure; HDL-C, HDL cholesterol; hs-CRP, high-sensitivity C-reactive protein; HOMA, homeostasis model assessment; LDL-C, LDL cholesterol; SBP, Systolic Blood Pressure: adjusted.

**^a^**Mean values ± SD at baseline and after 6 weeks and mean difference (95% confidence interval).

#### HOMA index

The HOMA index decreased from 4.8 ± 7.2 to 3.6 ± 2.7 in the iron-reduction group and from 4.5 ± 3.8 to 4.1 ± 3.6 in the control groups, resulting in a group difference of -0.7; 95% CI 2.1 to 0.6), but this was not significant (*P *= 0.29).

### Secondary outcome measures

Diastolic BP was significantly reduced (*P *< 0.001) by iron reduction. Furthermore, patients in this group had significant reductions in HR compared with the control group (*P *< 0.001) (Table 2).

Although the HOMA index did not indicate a significant increase in insulin sensitivity, plasma glucose and HbA1C were found to be significantly decreased in the iron-reduction group compared with the control group (*P *< 0.001 for both). No consistent changes in blood lipids could be verified in this study, but non-significant changes in low-density lipoprotein cholesterol (LDL-C) and HDL-C favoring the iron-reduction group resulted in a significant improvement in the LDL/HDL ratio (*P *< 0.01). Blood concentrations of adiponectin, hs-CRP and insulin were not changed by phlebotomy (Table 3).

We further analyzed the role of ferritin depletion and decreases in hemoglobin/hematocrit in the beneficial effect of blood-letting. Changes in SBP and serum ferritin concentration were significantly correlated with each other (Spearman's ρ = 0.41; *P *= 0.02). Furthermore, the non-significant improvement of HOMA index correlated with the decrease in serum ferritin concentration (ρ = 0.39; *P *= 0.03). No associations were found between changes in hematocrit, hemoglobin, red blood cell count, and outcomes.

### Safety

All patients tolerated the iron reduction by phlebotomy well, and no serious AEs occurred. In total, eight of the patients in the phlebotomy group reported mild AEs: four reported initial headaches that lasted for a few hours; three reported mild symptoms of dizziness, which did not last for more than 2 hours; and one patient had symptoms of fatigue for a few days. All patients of the phlebotomy group rated the procedure as tolerable, and the majority as very well tolerable (85%). All but one patient stated that they would be happy to undergo repeated phlebotomies.

## Discussion

This randomized clinical trial examined the effects of phlebotomy and controlled reduction of body iron in patients with METS. Reduction in iron stores resulted in substantial reduction in BP and improvement in glycemic control, LDL/HDL ratio, and resting HR at 6 weeks. No significant effect on insulin sensitivity was seen. Changes in BP and of insulin sensitivity correlated with decreases in serum ferritin concentration.

To our knowledge, there have been no randomized trials to date evaluating the effects of phlebotomy and iron reduction in METS or hypertension. However, an anti-hypertensive effect of repeated phlebotomy was described in an early uncontrolled study on 15 patients with hypertension resistant to triple antihypertensive medication [25]. In that study, phlebotomy lowered mean BP from 140.1 ± 12.2 mmHg to 123.8 ± 14.9 mmHg after 14 days. In another uncontrolled study, 12 patients with renal transplant and erythrocytosis received three phlebotomies of 500 ml over 6 weeks, which induced BP reductions from 153/95 mmHg to 139/85 mmHg [32].

Arterial hypertension, which affects about one-third of the adult population in the USA and Europe, causes enormous morbidity and mortality. Antihypertensive drug therapy is efficient and reduces morbidity and mortality, but is costly and causes undesirable AEs. In our study, we found a mean reduction in SBP of > 15 mmHg, indicating a clinically relevant effect. It has been estimated that that a 22% reduction in coronary events and a 41% reduction in stroke can be expected for a reduction in SBP of 10 mm Hg [33]. Furthermore, the observed reduction in resting HR of about 5 beats/min may translate to further cardiovascular risk reduction.

The effect of iron reduction on glucose metabolism was not consistent in our study. Whereas blood glucose and HbA1c were significantly reduced after iron-reduction therapy, there were no changes in insulin sensitivity or adiponectin secretion. In muscle, iron interferes with glucose uptake [34], and increased iron stores predict the development of diabetes in epidemiological studies [8-11]. A previous study found beneficial effects of phlebotomy in patients with type 2 diabetes with increased ferritin concentration [21]. In that study, patients 500 ml of blood removed three times at 2-weekly intervals, which resulted in a mean ferritin reduction from 500 to 230 ng/ml and significant reductions in HbA1c and HOMA index after 4 months. In a small safety study on blood donation, phlebotomy resulted ina significant decrease in serum glucose and blood lipids in patients with diabetes [22]. Iron reduction by phlebotomy also enhanced insulin sensitivity in patients with iron-induced insulin resistance and in carriers of the hemochromatosis gene [20]. Notably, in these studies, the amount of removed blood was larger than in our study and the study period was longer. Furthermore, we did not specify any predefined target ferritin level, and only a moderate ferritin reduction was achieved. Thus, it may be that the shorter duration of our study and the moderate reduction in body iron stores were not sufficient to improve insulin sensitivity. Moreover, the putative anti-diabetic effect of blood removal is likely to be more pronounced in patients with higher iron stores. The magnitude of effect might be smaller in an unselected population of patients with metabolic syndrome. In addition, measurement of insulin sensitivity by the HOMA method we used differs from intravenous methods. Given the assumption that the intravenous insulin tolerance test is more sensitive than the HOMA index, our trial might have been underpowered. Therefore, our results should be interpreted with caution regarding insulin sensitivity, and the clinical effect of iron reduction on insulin sensitivity in METS will need to be verified in larger trials.

We also found a modest effect of iron-reduction therapy on blood lipids, with an improved LDL/HDL ratio. In an earlier study, repeated phlebotomies decreased concentrations of triglycerides and total cholesterol [22]. In light of our findings, further evaluation of the effects of phlebotomy on blood lipids and metabolism seems warranted. Results from a controlled trial in patients with peripheral arterial disease found improved outcomes after iron reduction in younger and middle-aged subjects [35]. Our findings support a putative beneficial effect of iron reduction by phlebotomy on factors that can promote atherosclerosis.

The mechanisms responsible for the beneficial effects of venesection and blood letting in METS also need to be addressed. Based on our results, reductions in BP and HOMA index correlate significantly with ferritin reduction. Iron-catalyzed oxidative stress may have a negative effect on METS and BP through several mechanisms. In human monocytes of patients with hyperferritinemia associated with METS, manipulation of iron status induced cytokine release, and the degree of induction was correlated with carotid atherosclerosis [28]. Endothelium-dependent vasodilation is affected by oxidative stress, and thus iron-mediated oxidative stress may modulate vascular tone [26]. Generation of excess free oxygen radicals and loss of redox homeostasis have been related to insulin signaling, vascular tone, and associated cardiovascular functional abnormalities, with a putative dominant role of labile iron in the imbalance in redox homeostasis [4] However, some cardiovascular effects may also be related to the hemodynamic and hematologic consequences of phlebotomies. The reduction in blood volume caused by phlebotomy may lead to decreased extracellular fluid volume, peripheral resistance, and reductions in blood viscosity [36]. It was estimated that a 10% increase in hematocrit produces a 20% increase in blood viscosity, and that vasodilation or an increase of BP are required to compensate physiologically for the increased viscosity [37]. Thus, particularly in vessels with low vasodilatory capacity, phlebotomy might induce an additional antihypertensive effect by causing a reduction in viscosity.

The results of the present study should be interpreted in light of certain limitations inherent to the study design. Firstly, the study intervention was not blinded, and therefore we cannot exclude the possibility that non-specific effects contributed to the effectiveness of the intervention. We attempted to reduce the effects of disappointment in the control group by offering iron reduction therapy at the end of the study period, and we found that overall satisfaction with study participation was not different between groups. Secondly, we could not control for the lifestyle habits of our patients during the study. Patient self-reports and interviews and the unchanged BMI and waist circumference measurements did not indicate that relevant lifestyle changes had occurred in our study patients; however, modifications in diet and physical exercise over a short 6-week period could result in biochemical effects and reductions in BP without producing significant variations in weight and BMI. Thirdly, the definition of METS is not very specific, and our sample of patients was small; thus, our results may not be applicable to patients with METS in general. Finally, the study follow-up was limited to 6 weeks in this proof-of-concept study and, therefore, the results of the present trial should be regarded as preliminary. Further trials with longer observation periods should evaluate the long-term effects and potential rebound effects of phlebotomy therapy.

## Conclusion

In patients with METS, phlebotomy with moderate reduction of body iron stores lowered BP and resulted in improvements of markers of cardiovascular risk and glycemic control. We propose that adequately controlled phlebotomy could be considered as a cost-effective additional treatment option in METS. Furthermore, a beneficial health-related effect for blood donation might be a motivating factor to encourage more people to donate blood, providing public healthcare benefits as well.

## List of abbreviations

HOMA: homeostasis model assessment; METS: Metabolic syndrome; NAFLD: Non-alcoholic fatty liver disease.

## Competing interests

The authors declare that they have no competing interests.

## Authors' contributions

AM, RL, GD, MBB, KSH designed the study and contributed to acquisition of data. KSH, TR coordinated the study. Statistical analysis was carried out by RL. KSH, UK, AM, TR, RL interpreted the results. AM, UK, BB, GD, MBB revised the manuscript. All authors read and approved the final version of the manuscript.

## Pre-publication history

The pre-publication history for this paper can be accessed here:

http://www.biomedcentral.com/1741-7015/10/54/prepub
